# Clinical Phenotypes of Patients with Anti-DFS70/LEDGF Antibodies in a Routine ANA Referral Cohort

**DOI:** 10.1155/2013/703759

**Published:** 2013-02-07

**Authors:** Makoto Miyara, Roger Albesa, Jean-Luc Charuel, Mohamed El Amri, Marvin J. Fritzler, Pascale Ghillani-Dalbin, Zahir Amoura, Lucile Musset, Michael Mahler

**Affiliations:** ^1^Department of Immunology, (AP-HP) Pitié-Salpêtrière Hospital, Paris, France; ^2^Department of Research, INOVA Diagnostics Inc., 9900 Old Grove Road, San Diego, CA 32131-1638, USA; ^3^Faculty of Medicine, University of Calgary, Calgary, Canada; ^4^Department of Internal Medicine, (AP-HP) Pitié-Salpêtrière Hospital, Paris, France

## Abstract

*Objective*. To analyze the clinical value of anti-DFS70 antibodies in a cohort of patients undergoing routine antinuclear antibodies (ANAs) testing. *Methods*. Sera with a dense fine speckled (DFS) indirect immunofluorescence (IIF) pattern from 100 consecutive patients and 100 patients with other IIF patterns were tested for anti-DFS70 antibodies by a novel chemiluminescence immunoassay (CIA) and for ANA by ANA Screen ELISA (both INOVA). *Results*. Among the 100 patients with a DFS IIF pattern, 91% were anti-DFS70 positive by CIA compared to 3% in the comparator group (*P* < 0.0001). The CIA and IIF titers of anti-DFS antibodies were highly correlated (rho = 0.89). ANA by ELISA was positive in 35% of patients with the DFS IIF pattern as compared to 67% of patients with other patterns (*P* < 0.0001). Only 12.0% of patients with DFS pattern and 13.4% with DFS pattern and anti-DFS70 antibodies detected by CIA had systemic autoimmune rheumatic disease (SARD). Only 5/91 (5.5%) patients with anti-DFS70 antibodies had SARD and their sera were negative on the ANA Screen ELISA. *Conclusion*. Although anti-DFS70 antibodies cannot exclude the presence of SARD, the likelihood is significantly lower than in patients with other IIF patterns and should be included in test algorithms for ANA testing.

## 1. Introduction

The presence of antinuclear antibodies (ANAs), directed against intracellular antigens, is a hallmark of systemic autoimmune rheumatic diseases (SARDs) [1]. The indirect immunofluorescence (IIF) assay is among the most commonly used routine methods for ANA detection and was recently recommended as the screening test of choice by a study group of the American College of Rheumatology (ACR) [2]. Anti-dense fine speckles 70 (anti-DFS70) antibodies were initially identified as an ANA IIF pattern from a patient with interstitial cystitis [3], but were later associated with various other conditions (reviewed in [4]).

The typical DFS IIF staining pattern is recognized as uniformly distributed fine speckles throughout interphase nuclei and on metaphase chromatin [5]. The antigen was initially termed DFS70 according to the IIF pattern and the apparent molecular weight in immunoblot assays, but the primary target autoantigen was eventually identified as the lens epithelium-derived growth factor (LEDGF) [6] and more recently as the DNA binding transcription coactivator p75 (reviewed in [4]). DFS70/LEDGF is highly expressed in prostate tumour tissues [7] and has a number of physiological functions including serving as a cofactor for human immunodeficiency virus replication through an interaction with viral integrase [8].

Since the first description, anti-DFS70/LEDGF antibodies (hereafter referred to as anti-DFS70) have been reported in patients with a variety of chronic inflammatory conditions (reviewed in [4]), in cancer [7], and even in certain healthy individuals (HI) [9]. Dellavance et al. evaluated over 10,000 ANA positive samples by IIF and immunoblot and reported that anti-DFS70 antibodies were common among ANA-positive individuals with no evidence of SARD and that among autoimmune patients bearing this autoantibody, over half had evidence of autoimmune thyroiditis [10]. The highest prevalence of anti-DFS70 antibodies has been reported in patients with Vogt-Harada syndrome (66.7%) [11] and atopic dermatitis (AD, 30%) [3, 12] followed by HI (~10%) [4, 9], while its prevalence in SARD is significantly lower (~2-3%) [4]. Considering the prognostic and long term outcome of individuals with anti-DFS70 antibodies, it was recently reported that none of 40 HI with isolated anti-DFS70 reactivity developed a SARD within an average 4-year followup [13]. Therefore, it was suggested that the presence of isolated anti-DFS70 antibodies could be taken as strong evidence against a diagnosis of SARD such as systemic lupus erythematosus (SLE) [9, 13–15]. 

The low prevalence of anti-DFS70 antibodies in patients with SARD is interesting and represents a potentially important biomarker that can be clinically used to discriminate SARD from ANA-positive HI and/or other inflammatory conditions such as AD. The reasons underlying the observed relative low prevalence in SARD are unclear but may include the impact of therapeutic interventions (i.e., corticosteroids, immune suppression).

Since ANAs and related autoantibodies are generally considered useful biomarkers for SARD and are included in the classification criteria for SLE [16] and systemic sclerosis (SSc) [17], ANA testing on HEp-2 substrates outside a proper clinical framework may yield a sizable portion of ANA-positive individuals without consistent evidence of SARD, purportedly leading to inappropriate referrals to tertiary care specialists, as well as anxiety in patients and physicians alike [13] and, perhaps, inappropriate and potentially toxic therapies [18]. A proper understanding of the clinical relevance of the full spectrum of autoantibodies detected in a diagnostic laboratory becomes even more crucial because of compelling evidence that autoantibodies may precede the clinical onset of SARD for many years [19–21]. Therefore, the concept of utilizing anti-DFS70 antibodies as a diagnostic or prognostic discriminator of ANA positive subjects with and without SARD is appealing. Accordingly, the principal aims of this study were to determine the frequency of anti-DFS70 antibodies in samples showing a DFS staining pattern (against a control group with other patterns) and then to investigate the prevalence SARD in the two groups.

## 2. Materials and Methods

### 2.1. Clinically Defined Samples

Sera of 100 consecutive patients that were referred to a single hospital (Pitie-Salpetriere, Paris, France) with a DFS IIF pattern on HEp-2000 cell substrates (ImmunoConcepts) with titers higher or equal to 1:80 were the primary focus of this study. Sera of 100 patients with a positive ANA and other IIF patterns (homogeneous, *n* = 20; speckled, *n* = 20; homogeneous and speckled, *n* = 20; nucleolar, *n* = 20; speckled/nucleolar, *n* = 10; centromere, *n* = 10) were used as the comparator group. Samples were collected during an audit period from 7th of December 2011 to 25th of April 2012 and were from a spectrum of hospital departments. A diagnosis of SARD in patients was retrospectively analyzed by clinical chart review of medical records and was established according to the disease criteria for the respective disease and as described previously [22]. Patient identity was not disclosed and the data were anonymously used in accordance with the latest version of the Helsinki Declaration of human research ethics. Collection of patient samples was carried out according to local ethics committee regulations and ethical approval was obtained from the “CPP - Ile de France- VI” at the Pitié-Salpêtrière Hospital. No consent was needed from any patients involved in this study. It was a retrospective study, without modification in the followup of patients.

### 2.2. Indirect Immunofluorescence (IIF)

IIF was performed using HEp-2000 cells (ImmunoConcepts) using secondary anti-human IgG (H + L) supplied by and according to the manufacturer's instructions. The screening dilution was 1 : 80. Reading and interpretation of the IIF patterns was done by an experienced technologist on a Leica DM LB2, camera DFC 300FX, logiciel IM500, and a 40x objective. 

### 2.3. Chemiluminescent Anti-DFS70 Assay

All samples were tested for the presence of anti-DFS70 antibodies by a novel chemiluminescence immunoassay. The QUANTA Flash DFS70 assay is a novel (CIA) (research use only) that uses recombinant DFS70 (expressed in *E. coli*) coated onto paramagnetic beads and is designed for the BIO-FLASH instrument (Biokit s.a., Barcelona, Spain) [23]. The principles and protocols of the assay system have been previously described [24, 25]. In brief, the relative light units (RLUs) are proportional to the amount of isoluminol conjugate that is bound to the anti-human IgG, which in turn is proportional to the amount of anti-DFS70 antibodies bound to the antigen on the beads. Using a standard curve, all RLU values are converted into calculated units (CU). Samples with antibody titers above the analytical measuring range (AMR, 3.2–450.8 calculated units, CU, cut-off = 20 CUs) were prediluted 1 : 20 and retested to determine the exact anti-DFS70 antibody concentration.

### 2.4. Detection of Other Autoantibodies and ANA/DFS70 Antibody Score

Antinuclear antibodies were determined in all samples using the QUANTA Lite ANA Screen ELISA (INOVA), which is a semiquantitative ELISA for the detection of ANA. The antigens include chromatin (dsDNA and histones), Sm/RNP, SS-A/Ro60, SS-B/La, Scl-70/topoisomerase I, centromere, PCNA, Jo-1, mitochondria (M-2) and ribosomal-P protein, as well as extracts from HEp-2 nuclei and nucleoli [26, 27]. Samples were tested according to the manufacturer's instructions and were considered positive when values were larger or equal to 20 units. The ANA/DFS70 Score was calculated by dividing the ANA ELISA by the DFS70 CIA results and was expressed in calculated units (CUs).

### 2.5. Statistical Evaluation

Data was statistically evaluated using the Analyse-it software (Version 2.03; Analyse-it Software, Ltd., Leeds, UK). Mann-Whitney *U*-test and Fisher exact test were carried out to analyze the difference between groups. Cohen's *kappa* was used to analyze qualitative agreements. The BDT comparator was used to analyze differences between likelihood ratios as previously described [28, 29]. Spearman equation was used to analyze the agreement between the CIA and IIF titers. For all statistical tests *P* values < 0.05 were considered as significant.

## 3. Results

### 3.1. Anti-DFS70 Antibodies and ANA (by ELISA) in Samples with DFS and Other IIF ANA Patterns

**Figure 1:**
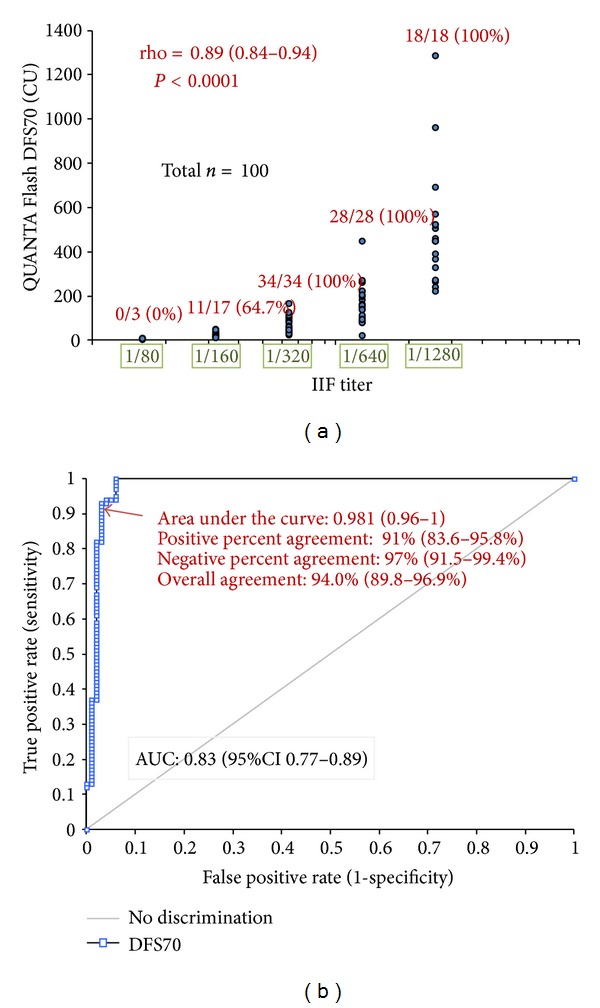
Correlation between dense fine speckled (DFS) pattern by indirect immunofluorescence (IIF) and anti-DFS70 antibodies measured by chemiluminescent immunoassay (CIA). (a) Correlation between the anti-DFS antibody titer by IIF and by QUANTA Flash DFS70. Excellent correlation between the anti-DFS antibody titers by IIF and by QUANTA Flash DFS70 was found using the samples showing the DFS speckled pattern (*n* = 100). Number and percent of the anti-DFS70 antibody positive samples are shown per titer group (cut-off = 20 CU). (b) Receiver operating characteristics (ROC) analysis comparing samples with DFS (*n* = 100) and other IIF ANA patterns (*n* = 100) by means of anti-DFS70 antibodies. Excellent discrimination between samples with DFS pattern and other patterns was observed as underlined by an area under the curve value of 0.981 (95% CI 0.960–1.000).

Among the 100 patients with DFS IIF pattern, 91% were anti-DFS70 positive by CIA compared to 3% in the comparator group with other IIF ANA patterns (*P* < 0.0001). The positive, negative, total percent agreements, and Cohen's *kappa* were 91.0% (95% Confidence interval; CI 83.6–95.8%), 97.0% (95% CI 91.5–99.4%), 94.0% (95% CI 89.8–96.9%), and 0.88 (95% CI 0.81–0.95), respectively (see Table 1). Receiver operating characteristics (ROC) analysis of anti-DFS70 antibodies demonstrated excellent discrimination between samples with DFS pattern (*n* = 100) and other IIF ANA patterns (*n* = 100) as underlined by an area under the curve value of 0.981 (95% CI 0.960–1.000) (Figure 1). Quantitative comparison of anti-DFS antibody titers by IIF and anti-DFS70 antibodies by CIA showed strong correlation (*P* < 0.0001, rho = 0.89, 95% CI 0.84–0.92). 

**Figure 2:**
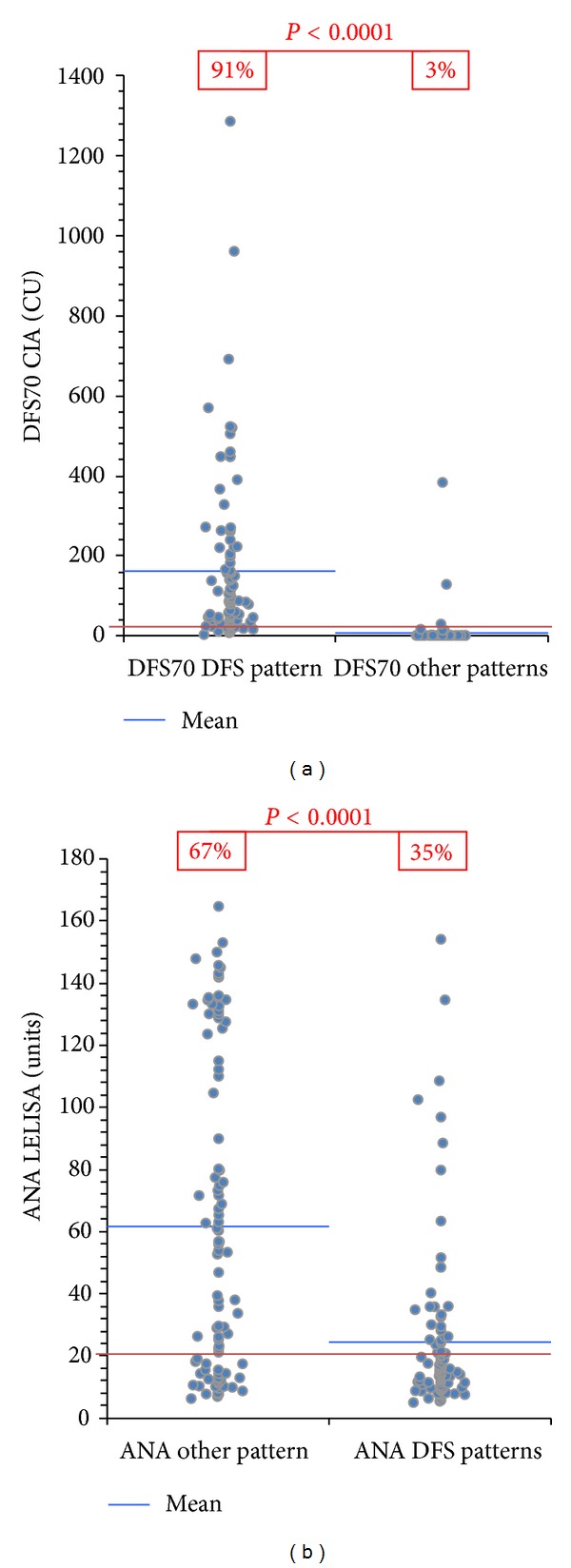
Antinuclear antibodies measured by ELISA and anti-DFS70 antibodies by chemiluminescent immunoassay (CIA) in samples with DFS pattern versus samples with other patterns. (a) Anti-DFS70 antibodies were significantly more prevalent (91.0% versus 3.0%) and their titers higher in samples with DFS pattern compared to samples with other patterns (*P* < 0.0001). (b) In contrast, antinuclear antibodies were significantly less prevalent (35.0% versus 67.0%) and their titers lower in samples with DFS pattern compared to samples with other patterns (*P* < 0.0001). Cut-off values are indicated by the red line.

The ANA Screen ELISA was positive in 67% of patients with other patterns versus 35% in patients with the DFS pattern (*P* < 0.0001). The positive, negative, total percent agreements, and Cohen's *Kappa* (ANA Screen ELISA and other patterns) were 67.0% (95% CI 56.9–76.1%), 65.0% (95% CI 54.8–74.3%), 66.0% (95% CI 59.0–72.5%), and 0.32 (95% CI 0.19–0.45), respectively (see Table 1). ANA titers were significantly higher in samples with other patterns compared to samples with the DFS pattern (*P* < 0.0001, Figure 2(b)). 

### 3.2. Differences in the Referring Physician Pattern of Samples with Dense Fine Speckled Pattern

The samples with the DFS pattern and other IIF ANA patterns were obtained from different referring clinical departments that included internal medicine/rheumatology, neurology, hepatology/gastroenterology, pulmonary diseases, ophthalmology, nephrology, intensive care, haematology, cardiology, infectious diseases, endocrinology, and otolaryngology). In the group with DFS pattern, 58 samples came from internal medicine/rheumatology versus 81 in the group with other patterns (*P* = 0.0007). In contrast, anti-DFS antibodies were more prevalent in samples from neurology (73.1% versus other patterns 34.6%; *P* = 0.0193) and hepatology (72.7% versus 36.4%, *P*= n.s.).

### 3.3. Clinical Association of the DFS Pattern and Anti-DFS70 Antibodies

The prevalence of SARD was significantly higher in the group with other ANA IIF patterns compared to the group with the DFS pattern (58% versus 12%, *P* < 0.0001) and to the group with a DFS pattern and confirmed anti-DFS70 antibodies (58% versus 13.4%, *P* < 0.0001). 15/94 (16.0%) patients with anti-DFS70 antibodies had SARD (13 SLE; 1 Sjögren's syndrome; 1 SSc). By comparison, only 5/94 (5.3%) patients with anti-DFS70 antibodies had SARD (4 SLE, 1 SSc) but were negative on the ANA Screen ELISA. Since an intended use of the DFS70 CIA is to confirm anti-DFS70 reactivity in samples showing the DFS pattern, we also calculated the clinical association in this subset of patients. 12/91 (13.4%) patients with DFS pattern and anti-DFS70 antibodies had SARD (10 SLE; 1 Sjögren's syndrome; 1 SSc). Only 5/91 (5.5%) patients with anti-DFS70 antibodies had SARD (4 SLE, 1 SSc) and were negative on the ANA Screen ELISA. The 3 anti-DFS70 positive patients in the control group had high titer (1/640–1/1280) homogeneous (*n* = 2) and homogeneous/speckled (*n* = 1) IIF ANA patterns and all had a diagnosis of SLE. 

**Figure 3:**
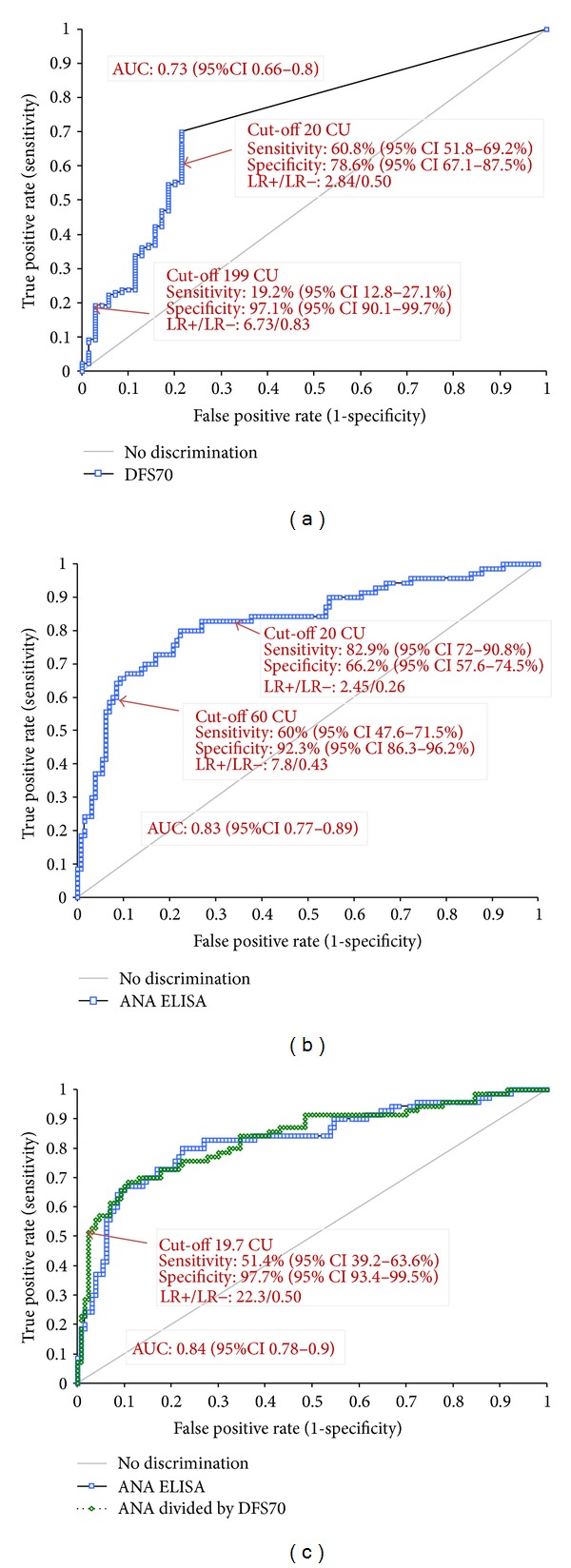
Discrimination between SARD and non-SARD patients using DFS70 and ANA by ELISA. (a) Differentiation of SARD versus non-SARD in all patients (*n* = 200) using DFS70. (b) Differentiation of SARD versus non-SARD in all patients (*n* = 200) using ANA Screen ELISA. (c) Differentiation of SARD versus non-SARD in all patients (*n* = 200) using ANA Screen ELISA and DFS70 Score (ANA ELISA divided by DFS70 CIA).

ROC analyses showed that anti-DFS70 antibodies discriminated between SARD and non-SARD patients (non-SARD patients having higher values, see Figure 3(a)) with an AUC of 0.73 (95% CI 0.66–0.80; *P* < 0.0001). At high titers (199 CU), 25/130 (19.2%) patients without SARD and 2/70 (2.9%) with SARD had anti-DFS70 antibodies. The likelihood ratios (LR+ and LR−) for non-SARD were 6.73 and 0.83, respectively. ANA by ELISA also discriminated between SARD and non-SARD patients (see Figure 3(b)) with an AUC of 0.83 (95% CI 0.77–0.89; *P* < 0.0001). Results are summarized in Table 2. At high titers (131.2 units), 17/70 (24.3%) patients with SARD and 3/130 (2.3%) were ANA ELISA positive. The likelihood ratios (LR+ and LR−) for SARD were 10.5 and 0.76, respectively, and thus more relevant compared to anti-DFS70 antibodies.

### 3.4. Algorithm of ANA ELISA and DFS70

Next we analyzed if the results derived from ANA Screen ELISA and from DFS70 CIA can be combined in a diagnostic score that improved the differentiation between SARD and non-SARD ANA IIF positive individuals. When using the results of the ANA/DFS70 Score for ROC comparative analysis we found a significantly improved discrimination between SARD patients and non-SARD patients (see Figure 3(c)) with an AUC of 0.84 (95% CI 0.78–0.90; *P* < 0.0001). The sensitivity and specificity at a selected cut-off were 51.4% and 97.7%. The LR+/− ratios for SARD was 22.3 and 0.50. When comparing the ANA/DFS70 Score with ANA ELISA at the same specificity (97.7%), the sensitivity of the ANA/DFS70 Score was significantly higher (51.4% versus 24.3%, *P* < 0.0001).

## 4. Discussion

Anti-DFS antibodies have been historically associated with interstitial cystitis [5] and atopic dermatitis [30], but they have also been described in various other diseases [4]. Although a distinctive clinical association is unreported, anti-DFS70 antibodies have been proposed as a useful biomarker for the exclusion of SARD [9, 14, 15, 23]. This suggestion has mainly been based on the observation that anti-DFS antibodies are more prevalent in HI than in SARD patients and that anti-DFS positive individuals did not develop SARD after clinical followup of four years [13]. Additional support for the hypothesis came from observations that approximately 30% of ANA positive samples from HI have anti-DFS70 antibodies [13, 31] as determined by IIF compared to 0% in ANA positive individuals with SARD. 

Anti-DFS70 antibodies have been reported in approximately 3% of SLE patients [14], but the detection of anti-DFS70 as detected by IIF may be problematic because these sera are usually accompanied by other antibodies such as anti-dsDNA, anti-SS-A/Ro, or anti-Sm, which may mask the DFS IIF staining pattern. In the SLE group reported by Muro et al. [14], 4/7 anti-DFS70 positive SLE were positive for anti-SS-A/Ro antibodies, 6/7 were also positive for dsDNA and 2/7 for anti-Sm. In a second study [23], the coexistence of other autoantibodies was similar: 5/7 anti-DFS70 positive SLE patients were positive for anti-dsDNA, and one for anti-Sm antibodies. Only 1/7 SLE patients with anti-DFS70 antibodies had no additional detectable autoantibodies. These data confirm that anti-DFS70 antibodies are rarely observed in SARD and when they are, they are usually accompanied by additional SARD related autoantibodies. In addition, no clinical difference between anti-DFS70 positive and negative SLE patients has been found. These data are consistent with our findings since 13.4% of anti-DFS70 positive but only 5.5% of anti-DFS70 positive/ANA ELISA negative patients had SARD.

The higher prevalence of anti-DFS70 antibodies in HI compared to SARD patients might support the hypothesis that these autoantibodies serve as protective [32] or indifferent or neutral effector [33] autoantibodies. However, further longitudinal studies are required to address this hypothesis. Despite the importance of these previous studies, a significant limitation is that they were based on selected serological cohorts and not on unselected patients for which an ANA test was requested. 

The prevalence of the DFS IIF pattern and anti-DFS70 antibodies has been reported to vary significantly [23]. One study reported that 172/21,512 (0.8%) of samples showed the typical DFS pattern by IIF [34] while another investigation showed that anti-DFS antibodies were present in as much as 12.3% of consecutive samples tested for ANA [10]. Although our study does not allow to. 

It has been reported that the frequency of anti-DFS70 antibodies in routine laboratories is within the range of other important SARD autoantibodies such as anti-dsDNA antibodies [35, 36]. In addition, it was found that virtually all samples with DFS pattern identified by IIF had anti-DFS70 antibodies by CIA and/or ELISA which is consistent with our findings. In our cohort we found a positive and negative percent agreement of 91.0% and 97.0%, respectively. However, since significant differences have been described between the staining patterns on HEp-2 cells from different manufacturers [35, 37], it remains unclear if the DFS IIF pattern can be recognized with similar accuracy using slides from a variety of manufacturers. Such variations might be attributed to the fixation methods, culture conditions, and/or other processes used for manufacturing the cell substrates [37]. Another variable to consider is the acumen of the laboratory personal in identifying the DFS pattern. Although previous data [23] indicate that the DFS pattern can be identified on slides from a number of ANA kit manufacturers, more samples need to be analyzed to arrive at a conclusion, especially since conflicting results have been published [37]. 

In our cohort of 200 ANA positive individuals, 130 had no evidence of SARD. Since a positive ANA test result is an important component in the triage and diagnosis of patients with possible SARD, ANA-HEp-2 testing outside a proper clinical framework may yield a sizable portion of ANA-positive apparently HI, causing concern and anxiety in patients and physicians [13], and may lead to prescribing inappropriate and potentially toxic therapeutics [18]. This concern becomes even more important with the recent knowledge that autoantibodies often precede the clinical onset of SARD by many years [19–21]. Hence, samples with DFS staining pattern identified by IIF should be tested for anti-DFS70 antibodies by a specific assay (i.e., ELISA or CIA) and the result should be included in the laboratory report. In addition, it is advisable that clinicians should not overinterpret positive ANA results in patients with anti-DFS70 antibodies alone but should focus on the clinical signs and symptoms complimented by the detection of other disease specific autoantibodies. The observation that ANA by ELISA (QUANTA Lite ANA Screen) is able to differentiate ANA positive patients with SARD from ANA positive non-SARD patients is interesting. It might be assumed that the ANA ELISA does not detect anti-DFS70 antibodies. The reason that the majority of samples containing anti-DFS70 antibodies are negative on the ANA Screen ELISA requires further investigations. All novel (optimized) cut-off of the DFS70 CIA, the ANA Screen ELISA and the ANA/DFS70 Score were established based on ROC analysis in our cohort of patients and have to be validated in further studies before clinically applied.

Historically, when the ANA HEp-2 test became available in the 1960s, predominantly rheumatologists and clinical immunologists ordered this test. With the eventual recognition that many other diseases with autoimmune features are also associated with ANAs, a broader range of clinical disciplines now order the ANA test. This change in test referral pattern has tremendous consequences for the posttest probability of disease since screening tests with limited specificity (such as IIF ANA) are strongly affected when the pretest probability in a given population decreases. Of note, in our cohort the prevalence of DFS versus other patterns was statistically different in two referral sources. In samples referred from internal medicine/rheumatology, the prevalence of other IIF patterns was higher than of the DFS pattern and in samples referred from neurology the difference was the opposite.

A significant limitation of our study is that most samples came from follow-up visits of the patients. However, since most individuals with ANAs including anti-DFS70 antibodies remain positive for many years [13] it can be speculated that our data is also relevant to the diagnostic setting. Further studies with diagnostic samples are needed to confirm our findings. 

Our data confirms previous observations that SARD is less prevalent in patients with a DFS pattern (and anti-DFS70 antibodies) than in patients with other patterns (i.e., homogeneous, speckled, homogeneous and speckled, nucleolar, speckled/nucleolar, centromere). Although the DFS pattern (and anti-DFS70 antibodies) cannot exclude the presence of SARD [38], the likelihood is significantly lower than with other patterns. Therefore, anti-DFS70 antibodies represent an important biomarker that can aid in the interpretation of positive ANA patients and, therefore, should be included in test algorithms for ANA testing. The optimal test algorithm might be laboratory specific being dependent on referral patterns for ANA testing.

## Figures and Tables

**Table 1:** Correlation between DFS and other IIF patterns and anti-DFS70 antibodies by ELISA and CIA.

Assay	DFS pattern	Other patterns	Positive % agreement*	Negative % agreement*	*P*
DFS70 CIA	91/100 (91.0%)	3/100 (3.0%)	91.0% (83.6–95.8%)	97.0% (91.5–99.4%)	*P* < 0.0001
ANA ELISA	35/100 (35.0%)	67/100 (67.0%)	67.0% (56.9–76.1%)	65.0% (54.8–74.3%)	*P* < 0.0001

*Positive and negative percent agreements were calculated based on the target cohort: For DFS70 CIA the target cohort is the group of samples with DFS pattern and the control cohort is the group of samples with other ANA patterns; for ANA ELISA the target cohort is the group of samples with other ANA patterns and the control cohort is the group of samples with DFS patterns.

**Table 2:** Discrimination of SARD and non-SARD using ANA ELISA, anti-DFS70 CIA, and ANA/DFS70 score (ANA divided by DFS70); different cut-offs.

	SARD	Non-SARD	Sensitivity	Specificity	LR+/LR−
DFS pattern^#^	12/70 (17.1%)	88/130 (67.7%)	67.7% (58.9–75.6%)	82.9% (72.0–90.8%)	2.57/0.25
QUANTA Flash DFS70^#^					
Cut-off 20 CU^&^	15/70 (21.4%)	79/130 (60.8%)	60.8% (51.8–69.2%)	78.6% (67.1–87.5%)	2.84/0.50
Cut-off 199 CU^+^	2/70 (2.9%)	25/130 (19.2%)	19.2% (12.8–27.1%)	97.1% (90.1–99.7%)	6.73/0.83
ANA Screen ELISA*					
Cut-off 20 units	58/70 (82.9%)	44/130 (33.8%)	82.9% (72.0–90.8%)	66.2% (57.3–74.2%)	2.45/0.26
Cut-off 60 units^+^	42/70 (60.0%)	10/130 (7.7%)	60.0% (47.6–71.5%)	92.3% (86.3–96.2%)	7.80/0.43
Cut-off 131.2 units^+^	17/70 (24.3%)	3/130 (2.3%)	24.3% (14.8–36.0%)	97.7% (93.4–99.5%)	10.5/0.76
ANA/DFS70 Score*					
ANA divided by DFS70 Cut-off 19.7 CU^+^	36/70 (51.4%)	3/130 (2.3%)	51.4% (39.2–63.6%)	97.7% (93.4–99.5%)	22.3/0.50

*Positive result (and LR+) considered indicative for SARD; ^#^positive result (and LR+) considered indicative for non-SARD; ^&^ cut off values were previously established; ^+^cutoff values were defined based on receiver operating characteristics (ROC) analysis.

## Abbreviations

ANA: Antinuclear antibody
DFS: Dense fine speckled
CIA: Chemiluminescent immunoassay
IBD: Inflammatory bowel disease
IIF: Indirect immunofluorescence
LEDGF: Lens derived epithelium growth factor
HI: Healthy individuals
LR: Likelihood ratio
RA: Rheumatoid arthritis
SARD: Systemic autoimmune rheumatic diseases
SSc: Systemic sclerosis
SLE: Systemic lupus erythematosus
SLEDAI: SLE disease activity index
SSc: Systemic sclerosis.
